# The effect of in utero ethinyl oestradiol exposure on the risk of cryptorchid testis and testicular teratoma in mice.

**DOI:** 10.1038/bjc.1990.337

**Published:** 1990-10

**Authors:** A. H. Walker, L. Bernstein, D. W. Warren, N. E. Warner, X. Zheng, B. E. Henderson

**Affiliations:** Department of Preventive Medicine, University of Southern California, Los Angeles 90033.

## Abstract

Epidemiological findings indicate that both cryptorchid testis and testicular germ cell cancer may be a result of high maternal oestrogen levels early in pregnancy. An experiment was conducted with a mouse strain (129 Sv-S1 C P) in which the males are susceptible to testicular teratomas to determine if the frequency of undescended testis and testicular teratoma in male offspring could be increased by administration of ethinyl oestradiol (EE) to pregnant mice before day 13 of gestation. This point in gestation marks the completion of the migration of germ cells to the gonadal ridge in mice and other studies with these mice have shown that the tumours are initiated in this critical time period. EE mixed with corn oil was administered by subcutaneous injection in doses of 0.02 (n = 76) and 0.2 (n = 102) mg kg-1 of body weight on gestational days 11 and 12. These mice were allowed to deliver their offspring and the males were killed at 15 days of age. Since the tumours are present from birth, this amount of time was allowed to permit the tumours to reach sufficient size for easy visual identification. Compared to controls (n = 63), who received corn oil alone, the treated mothers produced offspring who were significantly more likely to have a cryptorchid testis (P = 0.0001) and who had an increased risk, although not significant, of a testicular teratoma.


*Br. J. Cancer* (1990), **62**, 599–602

© Macmillan Press Ltd., 1990

### The effect of *in utero* ethinyl oestradiol exposure on the risk of cryptorchid testis and testicular teratoma in mice

A.H. Walker[1], L. Bernstein[1], D.W. Warren[2], N.E. Warner[3], X. Zheng[1] & B.E. Henderson[1,4]

[1]*Department of Preventive Medicine, University of Southern California, 1420 San Pablo St, PMB B-103(A), Los Angeles, CA 90033, USA;* [2]*Department of Physiology and Biophysics, and* [3]*Department of Pathology, University of Southern California, 2025 Zonal Ave., Los Angeles, CA 90033, USA; and* [4]*The Kenneth Norris Jr Cancer Center, 1441 Eastlake Ave., Los Angeles, CA 90033, USA.*

**Summary** Epidemiological findings indicate that both cryptorchid testis and testicular germ cell cancer may be a result of high maternal oestrogen levels early in pregnancy. An experiment was conducted with a mouse strain (129 Sv-S1 C P) in which the males are susceptible to testicular teratomas to determine if the frequency of undescended testis and testicular teratoma in male offspring could be increased by administration of ethinyl oestradiol (EE) to pregnant mice before day 13 of gestation. This point in gestation marks the completion of the migration of germ cells to the gonadal ridge in mice and other studies with these mice have shown that the tumours are initiated in this critical time period. EE mixed with corn oil was administered by subcutaneous injection in doses of 0.02 ($n = 76$) and 0.2 ($n = 102$) mg kg$^{-1}$ of body weight on gestational days 11 and 12. These mice were allowed to deliver their offspring and the males were killed at 15 days of age. Since the tumours are present from birth, this amount of time was allowed to permit the tumours to reach sufficient size for easy visual identification. Compared to controls ($n = 63$), who received corn oil alone, the treated mothers produced offspring who were significantly more likely to have a cryptorchid testis ($P = 0.0001$) and who had an increased risk, although not significant, of a testicular teratoma.

Crytorchidism is a known risk factor for human testicular cancer (Henderson *et al.*, 1979; Schottenfeld *et al.*, 1980; Depue *et al.*, 1983) and there is evidence that both conditions may share the common aetiological factor of high maternal oestrogen levels early in gestation. In animals, testicular maldescent has been experimentally produced by administering oestrogen during gestation (Jean, 1973; McLachlan *et al.*, 1975; Nomura & Kanzaki, 1977; Yasuda *et al.*, 1985). In humans, an increased frequency of cryptorchidism has been found in males who were exposed *in utero* to diethylstilbestrol (DES) (Cosgrove *et al.*, 1977; Whitehead & Leiter, 1981). Recently, it was shown that levels of free oestradiol measured in serum obtained during the first trimester of pregnancy of mothers bearing a cryptorchid son were significantly higher than those of control mothers (Bernstein *et al.*, 1988).

With regard to cancer of the testis (including seminoma and nonseminoma subtypes), epidemiological studies have indicated that high maternal oestrogen levels in the first trimester of pregnancy may increase the subsequent risk of germ cell tumours in male offspring (Henderson *et al.*, 1979; Depue *et al.*, 1983). Both exogenous and endogenous sources of oestrogen have been implicated (Henderson *et al.*, 1983; Bernstein *et al.*, 1986; Depue *et al.*, 1987). Certain sublines of the 129 J mouse strain have a relatively high spontaneous incidence of testicular teratomas. Embryonal carcinoma cells appear to be the stem cells of these tumours and give rise to the differentiated cell types often observed. As the mouse becomes older the incidence of the embryonal carcinoma cells is much lower, due most likely to their differentiation into normal cells. As is usually the case, all of the embryonal carcinoma cells eventually disappear and the tumour is benign (Stevens, 1982).

These tumours, which develop from the primordial germ cells, appear to be present by day 13 of gestation, by which time migration of germ cells to the gonadal ridge is completed in the mouse (Chiquone, 1954). At gestational day 12.5 the germ cells are located in the medullary portion of the genital ridge, but by gestational day 13.5 they are located within the seminiferous tubules (Stevens, 1966). The primordial germ cells appear to undergo an important period of maturation after 12 days of gestation that causes them to be resistant to teratocarcinogenesis (Stevens, 1982).

Experiments with these mice indicate that both genetic and environmental factors can affect the incidence of the testicular teratomas. Factors that suggest an environmental effect include an increased incidence of teratomas in second litters versus first ones, and in left versus right testes. The latter phenomenon may be due to the relocation of the spermatic artery caused by the introduction of the gene *iv* (*situs inversus viscerum*) into the 129 strain of mice (Stevens, 1982). In addition, changing the location of the genital ridge during embryonic development, before gestational day 13, can increase the incidence of teratomas (Stevens & Mackensen, 1961). When genital ridges from mouse fetuses of 12.5 days gestation were grafted on to adult testes of the same susceptible strain, teratomas developed in 82% of the grafts; whereas when the grafts were done at day 13.5 of gestation, teratomas occurred in only 8% of the grafts (Stevens, 1964).

By altering the hormonal environment of the fetal testis in this susceptible mouse strain through maternal exposure before gestational day 13, it may be possible to increase the incidence of fetal testicular teratomas without removing the genital ridge from its normal location. Further, it would be expected that the incidence of cryptorchid testes would be increased with this type of exposure. The following experiment was designed to test these hypotheses.

### Materials and methods

### *Animals and chemicals*

Inbred mice of the 129 Sv-S1 C P strain were provided by Dr Leroy Stevens from the Jackson Laboratory, Bar Harbor, Maine, USA. This strain was derived from the mouse subline, called 129/Sv, which has a spontaneous incidence of testicular teratomas of about 1–2% (Stevens, 1984). The 129 Sv-S1 C P strain has an incidence of teratomas of 7% among animals heterozygous for the S1 gene, which has been shown to affect the development of primordial germ cells. The C (pigment) and P (non-pinkeye) genes permit identification of the genotype of these animals from the phenotype. Animals with non-pigmented tail-tips are heterozygous for the S1 gene (S1/+), animals homozygous for the S1 gene

Correspondence: A.H. Walker.
Received 31 January 1990; and in revised form 1 May 1990.

(S1/S1) are white and non-viable, and animals without the S1 gene (+/+) have pigmented tail-tips (Stevens, 1984).

A breeding colony was developed at the University of Southern California School of Medicine to produce mice for this study. Seven-week-old females in the experimental groups were mated to males of the opposite tail tip colour in order to produce approximately equal numbers of offspring with non-pigmented and pigmented tail tips (i.e. with and without the S1 gene). Some older, previously untreated females who had had previous litters were also used in the experiment. After placement with the male (at a ratio of two females to one male per cage) the females were checked daily for the presence of a vaginal plug, and the day of that plug was considered to be day 0 of the pregnancy. Pregnant females were kept in individual cages and were fed mouse breeder pellets and water *ad libitum*.

Ethinyl oestradiol (EE) was selected for use in this experiment because it has 200 times the biological activity of oestradiol (E2) and is used in oral contraceptives. Fetal exposure to this compound may occur if a woman continues to take oral contraceptives after conception, before realising that she is pregnant. The doses chosen for this experiment (0.02 and 0.2 mg $kg^{-1}$ body weight) are equivalent to about 20 and 200 times the amount that a 120 pound (54 kg) woman would receive daily when taking an oral contraceptive containing 50 μg of EE. EE was purchased from Sigma Chemical Co. (St Louis, MO, USA), dissolved in DMSO, and diluted with corn oil.

### *Experimental design*

A total of 241 females with vaginal plugs were randomly allocated to one of three treatment groups: a vehicle control ($n = 63$), a low dose EE group (0.02 mg EE per kg body weight) ($n = 76$), or a high dose EE group (0.2 mg EE per kg body weight) ($n = 102$). Due to the inbred nature of the strain and also to the effects of EE itself, only 118 or 49% of these females produced at least one viable male offspring, with the highest percentage occurring in the control group (60%) and the lowest in the high dose group (39%). Ten females in each group who produced at least one viable male offspring were multiparous at the time of mating. The total number of male offspring obtained per treatment group was: control, 107; low dose, 109; and high dose, 115.

Because the transplant evidence suggests that teratoma induction occurs with greater frequency when the fetal testes are grafted before 13 days of gestation (Stevens, 1964), EE was administered before this time. An initial trial in which EE was administered on days 7–12 resulted in no offspring from 26 treated mothers. Thus, the exposure period was changed to days 11 and 12 in order to target the period of highest susceptibility and minimise the loss of offspring.

The EE was administered by subcutaneous injection using repeating dispensers which delivered 20 μl of the EE/corn oil solution with each injection. Controls were given 20 μl of corn oil in each injection. Concentrations of EE needed to deliver the required doses (0.02 and 0.2 mg $kg^{-1}$) were based on the mean weight of the animals in each treatment group obtained within 3 days of each injection.

The females were allowed to deliver their pups and male offspring were killed at 15 days of age by $CO_2$ asphyxiation. Since these tumours are present from birth (actually they have been observed in fetuses as early as 15 days of gestation: Stevens, 1962), offspring could have been killed earlier, but the 15 day period of time was selected to allow the tumours to reach sufficient size for easy visual identification. The testes were removed and examined for the presence of a tumour. Cryptorchidism of the testes was also noted and recorded.

### *Histology*

The testes were fixed in 10% buffered formalin solution and embedded in paraffin. Five μm sections were cut and stained with haematoxylin and eosin. All testes with suspected tumours, based on gross examination, were examined histologically as well as a 10% random sample of the testes which appeared normal.

### *Statistics*

The risk of crytorchid testis and testicular teratoma in each treatment group was compared to that of controls using odds ratio estimates. Mantel–Haenszel odds ratios were computed (Mantel & Haenszel, 1959) and adjusted for tail-tip pigment and litter number, as these factors affect the incidence of spontaneous tumours. Cornfield 95% confidence limits and adjusted tests of trend across treatment groups were also calculated (Breslow & Day, 1980).

### Results

### *Cryptorchid testis*

In the three experimental groups (i.e. the controls, low dose EE and high dose EE groups) 331 male offspring were obtained and a cryptorchid testis was observed in 37. The undescended testes were almost exclusively on the left side (only two were found to be on the right side) and 81% (30/37) occurred among the mice with pigmented tail-tips (those without the S1 gene).

A strong dose–response effect was found for the occurrence of cryptorchid testis as a result of EE exposure, after statistical adjustment for litter number and tail-tip pigment ($P = 0.0001$) (Table I). The adjusted odds ratios were 3.2 in the low dose group and 8.5 in the high dose group.

### *Tumour incidence*

Testicular teratomas were observed in 24 mice based on the gross examination. All but one were confirmed to be a teratoma after histological examination. Nearly 70% (16/23) of the teratomas were found on the left side. They ranged in appearance from a barely visible discoloured nodule to larger tumours measuring up to 0.9 cm in diameter which completely replaced the testis. No tumours were found in a 10% random sample of other pairs of testes which were grossly normal.

After statistically adjusting for the effects of tail-tip pigment and litter number, the risk of teratoma in the EE treated groups was more than double that for the corn oil control group (Table I), although these results were not statistically significant. No dose–response effect was observed ($P = 0.37$). Statistical adjustment for other factors, such as the tail-tip pigment of the father or occurrence of a cryptorchid testis, did not affect these results. The odds ratio obtained after combining both treatment groups and adjust-

**Table I** Adjusted odds ratios (OR) for development of a cryptorchid testis and testicular teratoma as a result of prenatal EE exposure

| *Treatment group* | *No./total* | *OR*[a] | *95% CI* |
|---|---|---|---|
| *Cryptorchid testis* | | | |
| Control | 4/107 | 1.0 | referent |
| Low dose EE[b] | 10/109 | 3.2 | (0.9–13.2) |
| High dose EE[c] | 23/115 | 8.5 | (2.3–28.8) |
| *P* value for trend = 0.0001 | | | |
| *Teratoma* | | | |
| Control | 4/107 | 1.0 | referent |
| Low dose EE[b] | 11/109 | 2.6 | (0.7–11.0) |
| High dose EE[c] | 8/115 | 2.1 | (0.5– 9.4) |
| *P* value for trend = 0.37 | | | |
| Control | 4/107 | 1.0 | referent |
| Low or high dose EE | 19/224 | 2.4 | (0.7– 9.1) |

[a]Adjusted for tail-tip pigment and litter number. [b]Low dose EE = 0.02 mg EE per kg on days 11 and 12. [c]High dose EE = 0.2 mg EE per kg on days 11 and 12.

ing for tail-tip pigment (of the offspring) and litter number was 2.4 (95% CI, 0.7–9.1).

Despite the fact that chance cannot be ruled out as an explanation for these findings, there was consistency in these results, with an increased incidence of tumours in the EE treated groups within the strata of litter number and tail-tip pigment (Table II). The increases due to EE treatment were most dramatic in the mice with non-pigmented tail-tips where the per cent with a teratoma increased from 3.1% among controls to 11.1% in first litter offspring exposed to either dose of EE, and from 14.3% to 23.8% in second litter mice.

*Joint occurrence of cryptorchid testis and teratoma*

Since the incidence rates for cryptorchid testis and for testicular teratoma are relatively low, the probability that both would occur in the same animal is quite low, if one assumes that they are independent events (i.e. that the joint probability equals the product of the two independent probabilities). There were four animals observed with both outcomes and all were in the high-dose group. After stratifying on the offspring's tail-tip pigment and the mother's litter number, the expected number of animals having both outcomes among the treated groups was 1.61, assuming these two outcomes are independent events (Table III). The largest excess of observed versus expected was among pigmented tail-tip mice from second litters (3 versus 0.56).

### Discussion

The results of this study show that administration of EE to pregnant mice of the 129 Sv-S1 C P strain before day 13 of gestation increases the risk of a cryptorchid testis in male offspring and suggest that the risk of a testicular teratoma may be increased as well. While the frequency of both outcomes appears to be related to oestrogen exposure, differences in the characteristics of animals developing these outcomes suggest that the aetiologic mechanisms may differ. The teratomas occurred more frequently in the non-pigmented tail-tip mice and in second litter mice; and no dose–response effect was seen. Cryptorchid testes were observed more often in the pigmented mice and appeared unrelated to litter number; and a strong dose–response effect was found. The joint occurrence of both outcomes was more frequent than expected, suggesting that both mechanisms may operate within the same mouse during this critical exposure period which marks the completion of the migration of the germ cells to the gonadal ridge.

Testicular descent is thought to be a two stage process with both stages affected by hormal mechanisms (Hutson & Donahoe, 1986). Exposure in early pregnancy would affect the first stage (the initial transabdominal phase) which is thought to be regulated by mullerian inhibiting substance. Oestrogens have been shown to inhibit mullerian inhibiting substance (Newbold *et al.*, 1984; Hutson *et al.*, 1985) and cause atrophy of the gubernaculum (Wensing, 1973; Grocock *et al.*, 1988).

The finding that elevated maternal oestrogen levels may increase the risk of a germ cell tumour is also supported by consideration of possible biological mechanisms. Regarding human testicular cancer, a mechanism has been suggested whereby high oestrogen levels adversely affect germ cells during their critical period of migration to the gonadal sites, which occurs during the fourth to sixth weeks of gestation (Henderson *et al.*, 1983). Later, during puberty and in young adulthood, it is hypothesised that tumour growth is promoted by exposure to high gonadotropin levels, resulting in the peak incidence of testis cancer which occurs between 20 and 40 years of age (Henderson *et al.*, 1983).

A possible mechanism for the adverse affect of oestrogen on germ cells has been demonstrated by Yasuda *et al.* (1986*a*, *b*) in studies using a non-susceptible mouse strain (jcl:ICR). In these studies pregnant females were exposed to the same concentrations of ethinyl oestradiol as used in the current study (i.e. 0.02 and 0.2 mg $kg^{-1}$ body weight) as well as 2.0 mg $kg^{-1}$ body weight during days 11–17 of gestation. The EE was mixed with olive oil and was administered by oral intubation; the fetuses were examined on gestational day 18.

Electron microscopic examination of fetal gondal tissue revealed accelerated prespermatogenesis. This was attributed to the increased ratio of dark to light Sertoli cells which was found as a result of EE exposure. The dark Sertoli cells function to increase proliferation of germ cells, while the light Sertoli cells arrest this process (Wartenburg, 1981). In normal prenatal development, the light cells eventually predominate, resulting in the arrest of germ cell proliferation until puberty. However, if the balance was shifted to the dark cells as a result of EE exposure, then germ cells may be induced to proliferate longer at an earlier stage.

Other work by Yasuda *et al.* (1986*b*) has indicated that such levels of EE exposure also result in decreased testosterone synthesis by Leydig cells, causing a suppression of spermatogenesis. Thus prenatal EE exposure appears to have a dual effect, resulting in accelerated prespermatogenesis due to the

**Table II** Percentage of mice with a teratoma by litter number and tail-tip pigment within treatment group

| Litter number and tail-tip pigment | Treatment group: Control % (No./total) | Low or high dose % (No./total) |
|---|---|---|
| First litter | | |
| Pigmented | 0.0 (0/48) | 1.2 ( 1/ 81) |
| Non-pigmented | 3.1 (1/32) | 11.1 (10/ 90) |
| Total | 1.2 (1/80) | 6.4 (11/171) |
| Second or later litter | | |
| Pigmented | 7.7 (1/13) | 9.4 ( 3/ 32) |
| Non-pigmented | 14.3 (2/14) | 23.8 ( 5/ 21) |
| Total | 11.1 (3/27) | 15.9 ( 8/ 53) |

**Table III** Number and percentage of mice with teratoma and cryptorchid testis, and observed *vs* expected number of both occurring in same animal, by tail-tip pigment and litter number within EE treated groups

| Tail-tip pigment and litter number | EE treated groups[a] Total *n* | Percentage with tumour | Percentage with cryptorchid testis | Number with both outcomes: observed | expected |
|---|---|---|---|---|---|
| Pigmented | | | | | |
| 1st litter | 81 | 1.2 | 25.9 | 0 | 0.25 |
| 2nd litter | 32 | 9.4 | 18.8 | 3 | 0.56 |
| Non-pigmented | | | | | |
| 1st litter | 90 | 11.1 | 5.6 | 1 | 0.56 |
| 2nd litter | 21 | 23.8 | 4.8 | 0 | 0.24 |
| Total | | | | 4 | 1.61 |

[a]Among the control animals no animal had both outcomes. Based on the incidence rates, 0.14 would have been expected.

imbalance caused in Sertoli cells and to disruption of spermatogenesis due to its effect on the Leydig cells (Yasuda *et al.*, 1986*a*).

Studies with mice susceptible to teratomas (129/Sv-ter) have found that strains with low numbers of primordial germ cells and a prolonged proliferative period have the highest incidence of teratoma formation (Noguchi & Stevens, 1982). It appears that germ cells are most susceptible to teratocarcinogenesis while in their highest proliferative period.

In summary, this experiment has demonstrated that maternal exposure to ethinyl oestradiol in the 129 Sv-S1 C P mouse strain before a critical time period during gestation can affect testicular descent and may affect the incidence of testicular teratomas. Each of these events may be associated with oestrogen exposure through different aetiological mechanisms. Further studies using this mouse model are necessary to determine if other maternal hormonal factors can affect the incidence of these outcomes and to understand the biological mechanisms related to them.

The authors would like to thank Dr Leroy Stevens of the Jackson Laboratory, Bar Harbor, Maine, for the donation of the mice of the 129 Sv-S1CP strain for this study. This work was supported by grants from the Division of Research Resources, National Institutes of Health (RR05356) and the American Cancer Society (SIG2A).

### References

BERNSTEIN, L., DEPUE, R.H., ROSS, R.K., JUDD, H.L., PIKE, M.C. & HENDERSON, B.E. (1986). Higher maternal levels of free estradiol in first compared to second pregnancy: a study of early gestational differences. *J. Natl Cancer Inst.*, **76**, 1035.

BERNSTEIN, L., PIKE, M.C., DEPUE, R.H., ROSS, R.K., MOORE, J.W. & HENDERSON, B.E. (1988). Maternal hormone levels in early gestation of cryptorchid males: a case-control study. *Br. J. Cancer*, **58**, 379.

BRESLOW, N.E. & DAY, N.E. (1980). *Statistical Methods in Cancer Research. Vol. 1: The Analysis of Case-control Studies.* IARC Scientific Publications no. 32. International Agency for Research on Cancer: Lyon.

CHIQUONE, A.D. (1954). The identification, origin, and migration of the primordial germ cells in the mouse embryo. *Anat. Rec.*, **118**, 135.

COSGROVE, M.D., BENTON, B. & HENDERSON, B.E. (1977). Male genitourinary abnormalities and maternal diethylstilbestrol. *J. Urol.*, **117**, 220.

DEPUE, R.H., BERNSTEIN, L., ROSS, R.K., JUDD, H.L. & HENDERSON, B.E. (1987). Hyperemesis gravidarum in relation to estradiol levels, pregnancy outcome, and other maternal factors: a seroepidemiologic study. *Am. J. Obstet. Gynecol.*, **156**, 1137.

DEPUE, R.H., PIKE, M.C. & HENDERSON, B.E. (1983). Estrogen exposure during gestation and risk of testicular cancer. *J. Natl Cancer Inst.*, **71**, 1151.

GROCOCK, C.A., CHARLTON, H.M. & PIKE, M.C. (1988). Role of the fetal pituitary in cryptorchidism induced by exogenous maternal oestrogen during pregnancy in mice. *J. Reprod. Fertil.*, **83**, 295.

HENDERSON, B.E., BENTON, B., JING, J., YU, M.C. & PIKE, M.C. (1979). Risk factors for cancer of the testis in young men. *Int. J. Cancer*, **23**, 598.

HENDERSON, B.E., ROSS, R.K., PIKE, M.C. & DEPUE, R.H. (1983). Epidemiology of testis cancer. In *Urological Cancer*, Skinner, D.G. (ed.). Grune and Stratton: New York.

HUTSON, J.M. & DONAHOE, P.K. (1986). The hormonal control of testicular descent. *Endocr. Rev.*, **7**, 270.

HUTSON, J.M., DONAHOE, P.K. & MACLAUGHLIN, D.T. (1985). Steriod modulation of mullerian duct regression in the chick embryo. *Gen. Comp. Endocrinol.*, **57**, 88.

JEAN, C. (1973). Croissance et structure des testicules cryptorchides chez les souris nees de meres truitees a l'obstetractiol parchant la gestation. *Ann. Endocrinol.* (*Paris*), **34**, 669.

MANTEL, N. & HAENSZEL, W. (1959). Statistical aspects of the analysis of data from retrospective studies of disease. *J. Natl Cancer Inst.*, **22**, 719.

MCLACHLAN, J.A., NEWBOLD, R.R. & BULLOCK, B. (1975). Reproductive tract lesions in male mice exposed prenatally to diethylstilbestrol. *Science*, **190**, 991.

NEWBOLD, R.R., SUZUKI, Y. & MCLACHLAN, J.A. (1984). Mullerian duct maintenance in heterotypic organ culture after *in vivo* exposure to diethylstilbestrol. *Endocrinology*, **115**, 1863.

NOGUCHI, T. & STEVENS, L.C. (1982). Primordial germ cell proliferation in fetal testes in mouse strains with high and low incidences of congenital testicular teratomas. *J. Natl Cancer Inst.*, **69**, 907.

NOMURA, T. & KANZAKI, T. (1977). Induction of urogenital anomalies and some tumors in the progeny of mice receiving diethylstilbestrol during pregnancy. *Cancer Res.*, **37**, 1099.

SCHOTTENFELD, D., WARSHAUER, M.E., SHERLOCK, S., ZAUBER, A.G., LEDER, M. & PAYNE, R. (1980). The epidemiology of testicular cancer in young adults. *Am. J. Epidemiol.*, **112**, 232.

STEVENS, L.C. (1964). Experimental production of testicular teratomas in mice. *Proc. Natl Acad Sci USA*, **52**, 654.

STEVENS, L.C. (1966). Development of resistance to teratocarcinogenesis by primordial germ cells in mice. *J. Natl Cancer Inst.*, **37**, 859.

STEVENS, L.C. (1982). Teratocarcinogenesis and parthenogenesis. In *The Mouse in Biomedical Research, Vol IV, Experimental Biology and Oncology*, Foster, H.L., Small, J.D. & Fox, J.G. (eds). p. 161. Academic Press: New York.

STEVENS, L.C. (1984). Spontaneous and experimentally induced testicular teratomas in mice. *Cell Differentiation*, **15**, 69.

STEVENS, L.C. & MACKENSEN, J.A. (1961). Genetic and environmental influences on teratocarcinogenesis in mice. *J. Natl Cancer Inst.*, **27**, 443.

WARTENBURG, H. (1981). Differentiation and development of the testes. In *The Testis*, Burger, H. & de Krester, D. (eds). Raven Press: New York.

WENSING, C.J.G. (1973). Testicular descent in some domestic animals. III: Search for the factors that regulate the gubernacular reaction. *Proc. Kon. Ned. Akad. Wetensch.*, **76**, 196.

WHITEHEAD, D.E. & LEITER, E. (1981). Genital abnormalities and abnormal semen analyses in male patients exposed to diethylstilbestrol *in utero*. *J. Urol.*, **125**, 47.

YASUDA, Y., KIHARA, T., TANIMURA, T. & NISHIMURA, H. (1985). Gonadal dysgenesis induced by prenatal exposure to ethinyl estradiol in mice. *Teratology*, **21**, 219.

YASUDA, Y., KONISHI, H., MATUSO, T. & TANIMURA, T. (1986*a*). Accelerated differentiation in seminiferous tubules of fetal mice prenatially exposed to ethinyl estradiol. *Anat. Embryol.*, **174**, 289.

YASUDA, Y., KONISHI, H. & TANIMURA, T. (1986*b*). Leydig cell hyperplasia in fetal mice treated transplacentally with ethinyl estradiol. *Teratology*, **33**, 281.


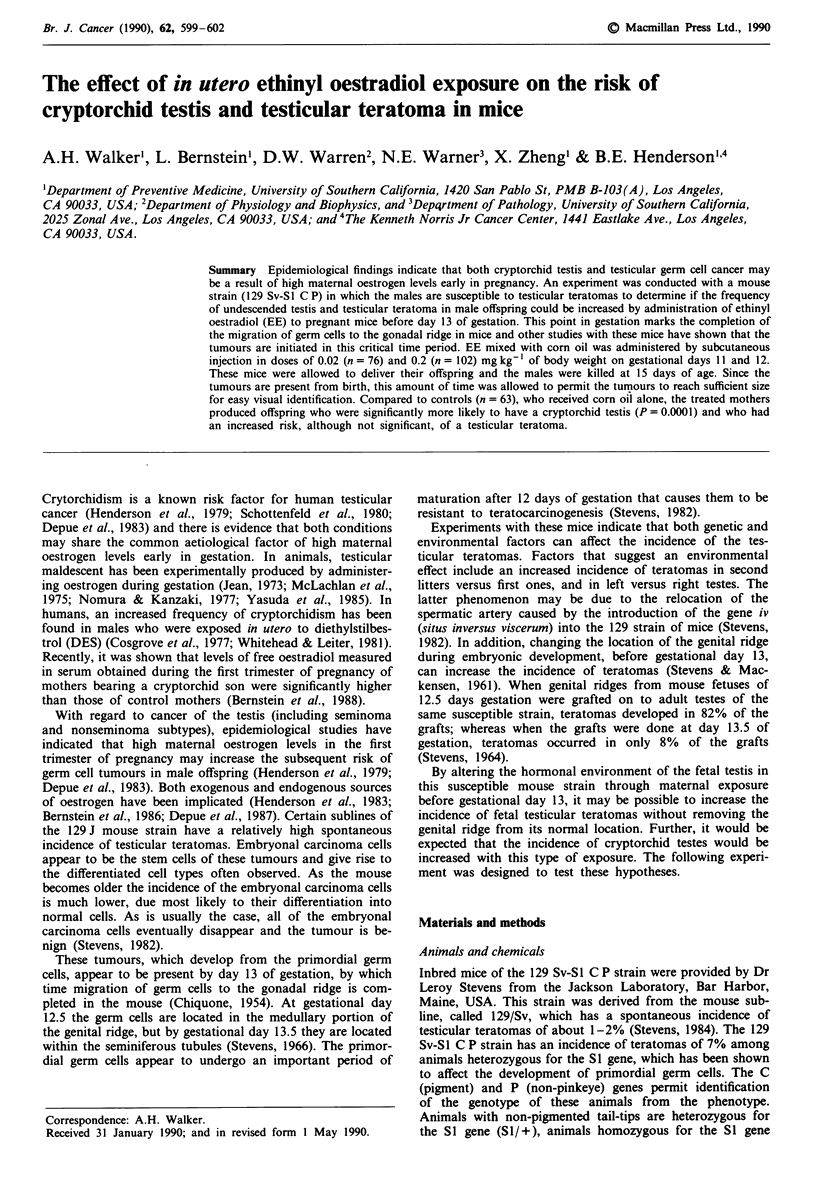

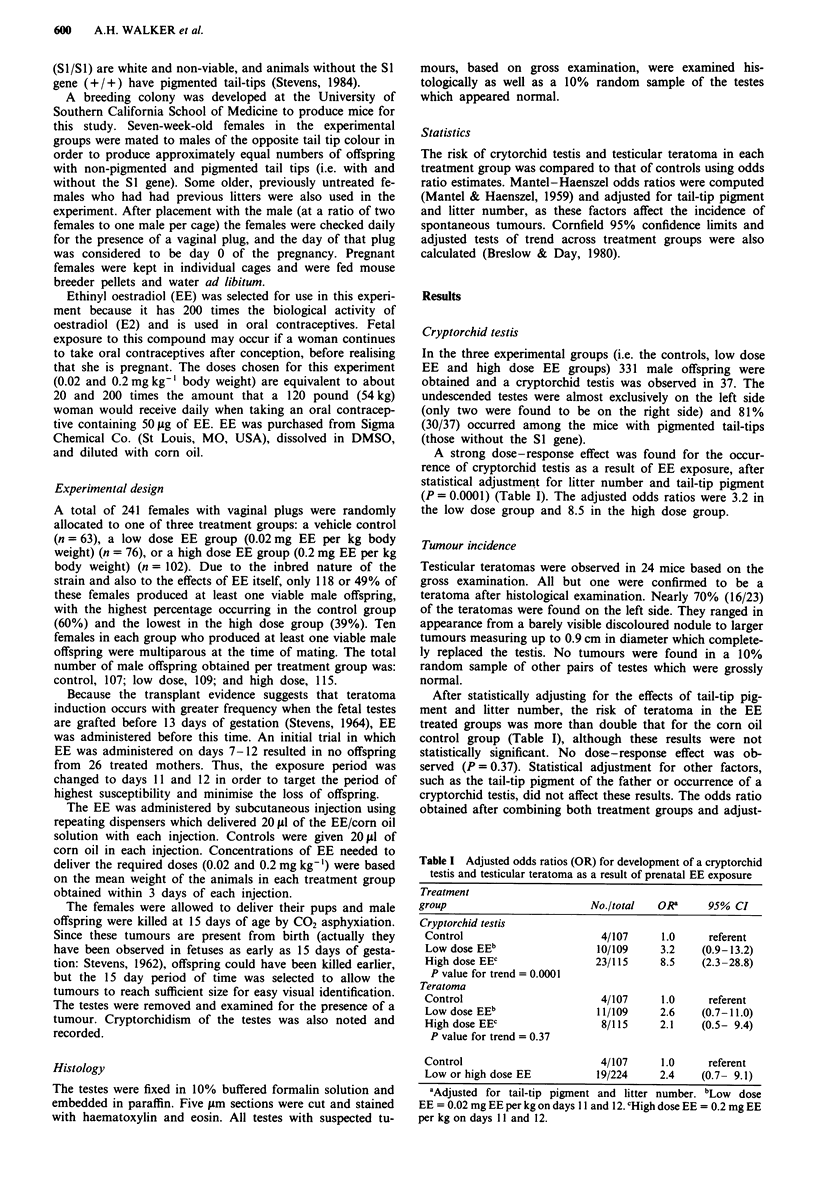

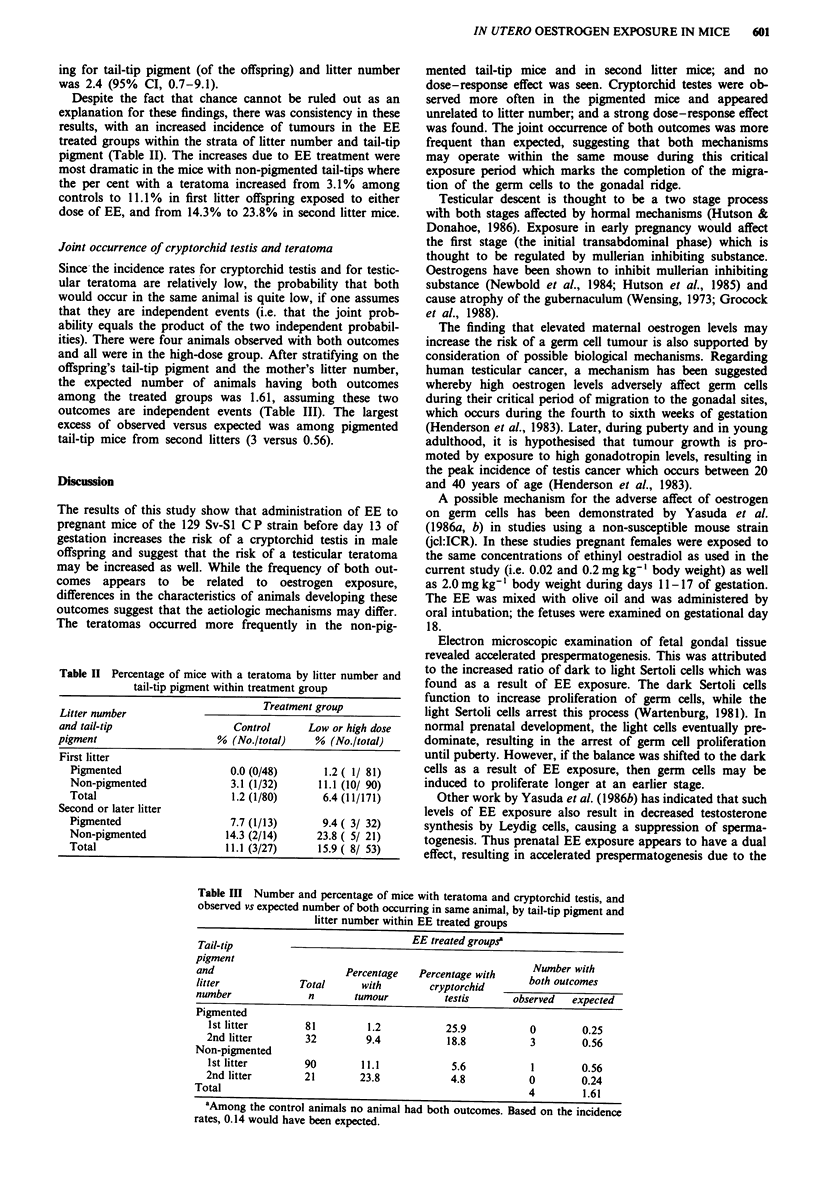

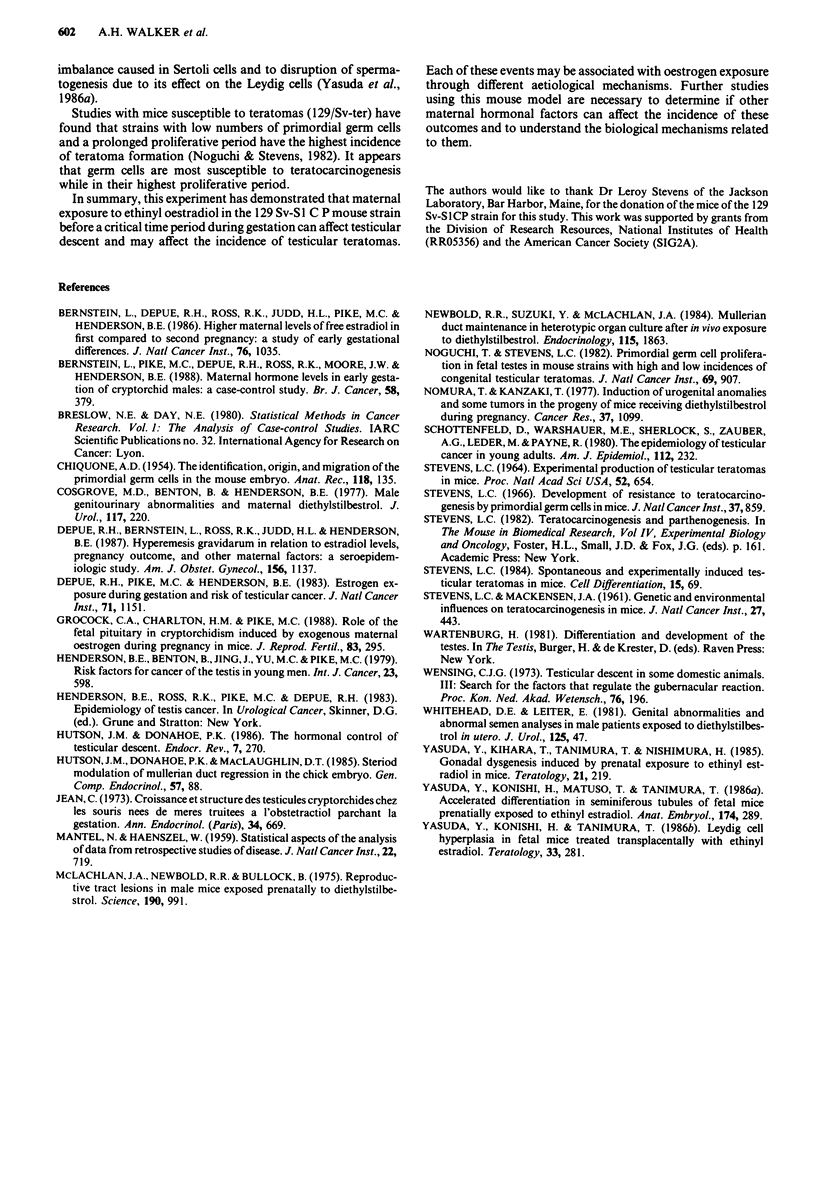


## References

[OCR_00479] Bernstein L., Depue R. H., Ross R. K., Judd H. L., Pike M. C., Henderson B. E. (1986). Higher maternal levels of free estradiol in first compared to second pregnancy: early gestational differences.. J Natl Cancer Inst.

[OCR_00485] Bernstein L., Pike M. C., Depue R. H., Ross R. K., Moore J. W., Henderson B. E. (1988). Maternal hormone levels in early gestation of cryptorchid males: a case-control study.. Br J Cancer.

[OCR_00497] CHIQUOINE A. D. (1954). The identification, origin, and migration of the primordial germ cells in the mouse embryo.. Anat Rec.

[OCR_00501] Coscrove M. D., Benton B., Henderson B. E. (1977). Male genitourinary abnormalities and maternal diethylstilbestrol.. J Urol.

[OCR_00506] Depue R. H., Bernstein L., Ross R. K., Judd H. L., Henderson B. E. (1987). Hyperemesis gravidarum in relation to estradiol levels, pregnancy outcome, and other maternal factors: a seroepidemiologic study.. Am J Obstet Gynecol.

[OCR_00512] Depue R. H., Pike M. C., Henderson B. E. (1983). Estrogen exposure during gestation and risk of testicular cancer.. J Natl Cancer Inst.

[OCR_00517] Grocock C. A., Charlton H. M., Pike M. C. (1988). Role of the fetal pituitary in cryptorchidism induced by exogenous maternal oestrogen during pregnancy in mice.. J Reprod Fertil.

[OCR_00522] Henderson B. E., Benton B., Jing J., Yu M. C., Pike M. C. (1979). Risk factors for cancer of the testis in young men.. Int J Cancer.

[OCR_00536] Hutson J. M., Donahoe P. K., MacLaughlin D. T. (1985). Steroid modulation of Mullerian duct regression in the chick embryo.. Gen Comp Endocrinol.

[OCR_00532] Hutson J. M., Donahoe P. K. (1986). The hormonal control of testicular descent.. Endocr Rev.

[OCR_00546] MANTEL N., HAENSZEL W. (1959). Statistical aspects of the analysis of data from retrospective studies of disease.. J Natl Cancer Inst.

[OCR_00551] McLachlan J. A., Newbold R. R., Bullock B. (1975). Reproductive tract lesions in male mice exposed prenatally to diethylstilbestrol.. Science.

[OCR_00556] Newbold R. R., Suzuki Y., McLachlan J. A. (1984). Müllerian duct maintenance in heterotypic organ culture after in vivo exposure to diethylstilbestrol.. Endocrinology.

[OCR_00561] Noguchi T., Stevens L. C. (1982). Primordial germ cell proliferation in fetal testes in mouse strains with high and low incidences of congenital testicular teratomas.. J Natl Cancer Inst.

[OCR_00566] Nomura T., Kanzaki T. (1977). Induction of urogenital anomalies and some tumors in the progeny of mice receiving diethylstilbestrol during pregnancy.. Cancer Res.

[OCR_00576] STEVENS L. C. (1964). EXPERIMENTAL PRODUCTION OF TESTICULAR TERATOMAS IN MICE.. Proc Natl Acad Sci U S A.

[OCR_00571] Schottenfeld D., Warshauer M. E., Sherlock S., Zauber A. G., Leder M., Payne R. (1980). The epidemiology of testicular cancer in young adults.. Am J Epidemiol.

[OCR_00580] Stevens L. C. (1966). Development of resistance to teratocarcinogenesis by primordial germ cells in mice.. J Natl Cancer Inst.

[OCR_00589] Stevens L. C. (1984). Spontaneous and experimentally induced testicular teratomas in mice.. Cell Differ.

[OCR_00603] Wensing C. J. (1973). Testicular descent in some domestic mammals. 3. Search for the factors that regulate the gubernacular reaction.. Proc K Ned Akad Wet C.

[OCR_00608] Whitehead E. D., Leiter E. (1981). Genital abnormalities and abnormal semen analyses in male patients exposed to diethylstilbestrol in utero.. J Urol.

[OCR_00613] Yasuda Y., Kihara T., Tanimura T., Nishimura H. (1985). Gonadal dysgenesis induced by prenatal exposure to ethinyl estradiol in mice.. Teratology.

[OCR_00618] Yasuda Y., Konishi H., Matuso T., Tanimura T. (1986). Accelerated differentiation in seminiferous tubules of fetal mice prenatally exposed to ethinyl estradiol.. Anat Embryol (Berl).

[OCR_00622] Yasuda Y., Konishi H., Tanimura T. (1986). Leydig cell hyperplasia in fetal mice treated transplacentally with ethinyl estradiol.. Teratology.

